# Cell surface RNAs define a new interface for immune recognition

**DOI:** 10.3389/fimmu.2026.1889356

**Published:** 2026-07-09

**Authors:** Xinming Zhang, Jiahao Liao, Yuduo Chang, Xiangli Shao, Yuan Ma

**Affiliations:** 1 Department of Chemistry, Rice University, Houston, TX, United States; 2 SynthX Center, Rice University, Houston, TX, United States; 3 Center for Nanoscale Imaging Sciences, Rice University, Houston, TX, United States

**Keywords:** cell surface RNA, glycoRNA, RNA, immunity, receptor, RNA recognition and immunity

## Abstract

Cell surface glycoRNAs have recently emerged as an unexpected class of RNA-glycan conjugates. Their discovery challenges the long-standing view that RNA is confined to intracellular compartments. These small noncoding RNAs, modified with N- or O-linked glycans and displayed on the outer leaflet of the plasma membrane, create a unique molecular interface recognizable by both glycan-binding and nucleic acid-sensing immune receptors. Recent studies suggest that glycoRNAs engage multiple immune receptor families, including P-selectin, Siglecs, Toll-like receptors (TLRs), and lectins, to influence neutrophil trafficking, immune activation, and tolerance. For instance, interactions between P-selectin and glycoRNAs have been implicated in neutrophil recruitment. In this Mini-Review, we summarize current knowledge of the molecular features of glycoRNAs and their emerging interfaces with immune receptors and discuss how glycoRNAs may represent a new layer of immune regulation at the cell surface.

## 1 Introduction

The identification of RNA molecules on the outer surface of mammalian cells challenges the long-standing view that RNA functions exclusively within intracellular compartments (1). This discovery also reveals an unexpected molecular interface for immune recognition (2). Traditionally, RNA has been considered a molecule confined to the nucleus and cytoplasm, where it regulates gene expression (3), protein synthesis (4), and cellular homeostasis (5). However, recent studies have uncovered RNA species associated with the plasma membrane, including membrane-associated extracellular RNAs (6) and glycoRNAs (1), suggesting that RNA may participate in extracellular molecular interactions and cell-cell communication (7–10). These findings expand the spatial and functional landscape of RNA biology and raise new questions regarding RNA-mediated recognition at cellular interfaces (11–13).

A key advance in this emerging field was the discovery of glycosylated RNAs, termed glycoRNAs, which represent a previously unrecognized class of RNA-containing glycoconjugates (1). The identification of glycoRNAs was enabled by glycan metabolic labeling combined with bioorthogonal click chemistry, an approach initially developed for profiling protein-associated glycans. In this strategy, azide-functionalized sugars are metabolically incorporated into cells, followed by RNA isolation and copper-free click chemistry to label glycans. Notably, azide-dependent signals were detected in highly purified RNA fractions and remained resistant to proteinase K digestion, indicating that these signals originated from glycan-modified RNAs rather than protein contaminants. Subsequent enrichment and sequencing analyses demonstrated that these species correspond to small noncoding RNAs covalently modified with glycans.

## 2 Molecular features of cell surface glycoRNAs

**Figure 1:**
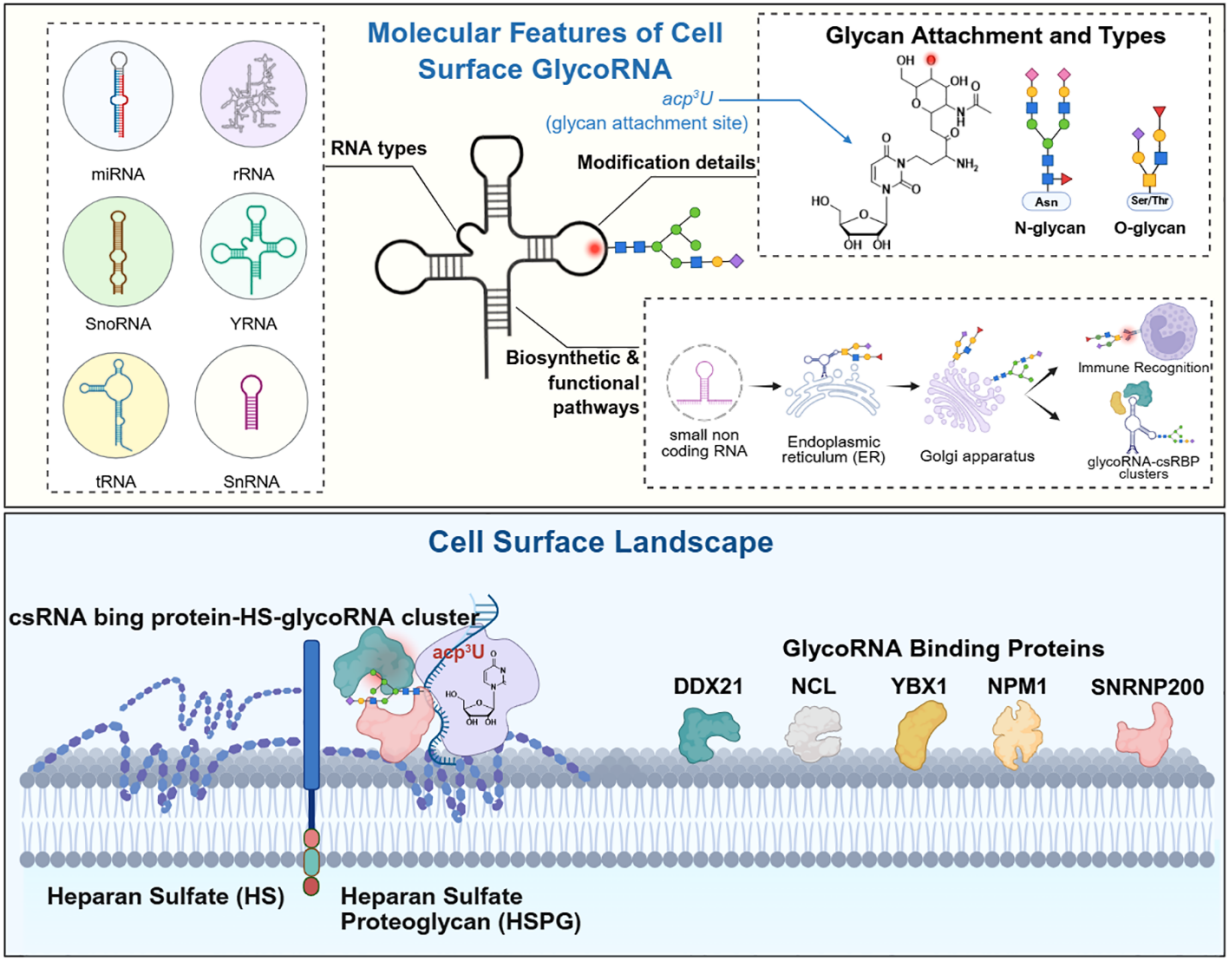
Illustration of the molecular features and functional landscape of cell surface glycoRNA, featuring diverse RNA types (e.g., tRNA, YRNA) modified with N/O-glycans via acp^3^U sites. The schematic depicts their biosynthesis through the ER-Golgi secretory pathway and subsequent localization at the plasma membrane. Here, glycoRNA interacts with HSPG and specific binding proteins like DDX21 and YBX1. These interactions facilitate critical extracellular processes, including immune recognition and the formation of glycoRNA-csRBPs clusters, highlighting their role in mediating complex cellular communication and surface signaling. Created with BioRender.com under a BioRender publication license.

At the molecular level, glycoRNAs exhibit several defining features. Their RNA backbone is predominantly composed of small noncoding RNAs, including Y RNAs, small nuclear RNAs (snRNAs), ribosomal RNAs (rRNAs), and small nucleolar RNAs (snoRNAs) (1, 9). Glycosylation occurs through the modified nucleotide 3-amino-3-carboxypropyl uridine (acp^3^U) (14), which provides a linkage site for N-glycans, and these RNAs can further assemble with cell surface RNA-binding proteins (csRBPs) into nanoscale extracellular complexes (15). In addition to their RNA components, glycoRNAs carry both N-linked and O-linked glycans that resemble those found on glycoproteins but display distinct patterns of presentation (16–18). Importantly, glycoRNAs localize to the outer leaflet of the plasma membrane, where they colocalize with csRBPs and heparan sulfate proteoglycans (HSPGs) (19, 20), forming organized molecular assemblies within the glycocalyx (**Figure 1**). This extracellular localization represents a significant departure from the traditional view that RNA molecules are restricted to intracellular environments.

**Figure 2:**
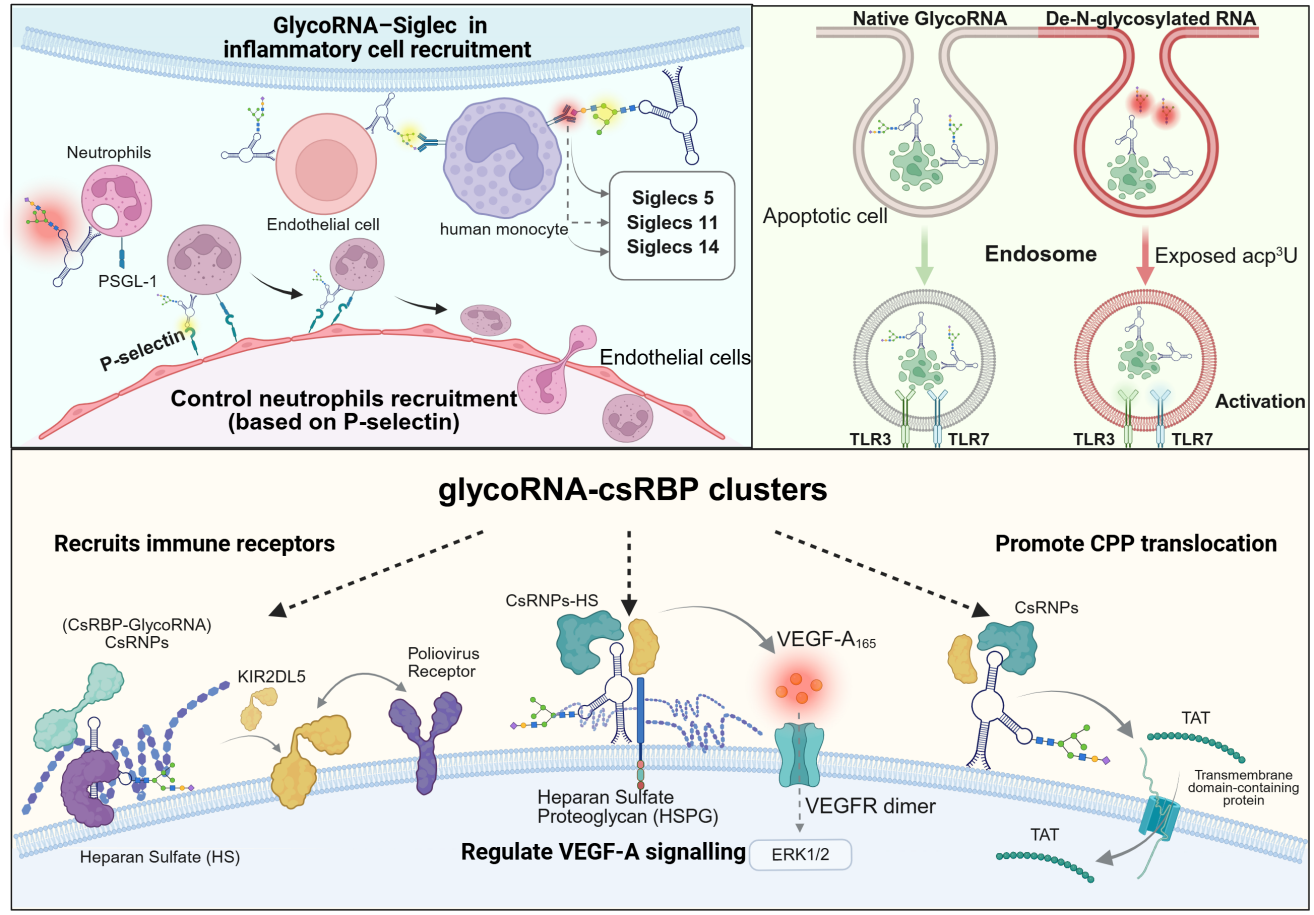
The diverse immunological roles of glycoRNA through specific receptor interactions. In cell recruitment, glycoRNA independently binds Siglecs (5/11/14) and P-selectin. Within endosomes, de-glycosylated RNAs expose acp^3^U sites to activate Toll-like receptors (TLR3/7), triggering innate immune responses. Additionally, surface glycoRNA-csRBPs clusters facilitate the recruitment of immune receptors like KIR2DL5, and promote their binding to the poliovirus receptor. Furthermore, glycoRNA-HSPG structures directly regulate angiogenesis by binding to the heparin-sulfate binding domain of VEGF-A. These csRBPs co-assemble with glycoRNA to form specific nanoscale cluster domains, which serve as essential gateways for CPP-mediated entry. Collectively, these interactions establish glycoRNA as a pivotal mediator of extracellular recognition and multifaceted immune signaling. Created with BioRender.com under a BioRender publication license.

The integration of RNA sequences, glycan structures, and associated protein scaffolds suggests that glycoRNAs form composite molecular platforms capable of mediating diverse ligand-receptor interactions at the cell surface. Given that immune surveillance relies on molecular recognition of glycans and nucleic acids, glycoRNAs may function as previously unappreciated ligands that bridge these recognition systems. Emerging evidence indicates that glycoRNAs participate in processes such as leukocyte recruitment, modulation of inflammatory signaling, and regulation of innate immune responses (2, 9, 21). In the following sections, we focus on how these molecular features enable glycoRNAs to engage immune receptors and shape immune communication (**Figure 2**; **Table 1**).

**Table 1:** Chemical biology tools for dissecting the receptors of glycoRNAs.

Receptor	GlycoRNA enrichment	Proximity labeling	Imaging methods	Genetic methods
P-selectin (9)	Ac4ManNAz-Biotin- Streptavidin-based pulldown WGA-lectin-based pulldown	N/A	Immunofluorescence confocal imaging	*Sidt1* and *Sidt2* Knock down P-selectin knockout
Siglecs (1, 10, 19)	Ac4ManNAz-labelling-based enrichment	N/A	Immunofluorescence confocal imaging Flow cytometry-based imaging	Genome-wide CRISPR-Cas9 gene-knockout
TLR (2)	Ac4ManNAz-labelling-based enrichment	N/A	N/A	*DTWD2* knockout TLR3 and TLR7 knockout
csRBPs (15)	Ac4ManNAz-labelling-based enrichment	HRP-biotin-phenol Biotin-aniline	Single-molecule super-resolution microscopy Immunofluorescence confocal imaging	Certain csRBPs knock down
csRNA-HS-csRBPs (20)	TLR7 probe-based enrichment	TLR-Fc	Immunofluorescence confocal imaging	Genome-wide CRISPR-Cas9 gene knockout
GlycoRNA-HSPG (19)	Siglec-11/VEGF-immunoprecipitation-based pulldown	Biotin-aniline	Immunofluorescence confocal imaging Flow cytometry-based imaging	Genome-wide CRISPR-Cas9 gene Knockout *HS2ST1* and *HS6ST1* Knockout

N/A, not applicable.

## 3 Molecular interfaces between glycoRNAs and immune receptors

### 3.1 P-selectin and leukocyte trafficking

P-selectin, also named Selp, is a lectin-type adhesion receptor encoded by the SELP gene, predominantly expressed by endothelial cells (ECs) and platelets (22, 23). It rapidly translocates to the surface of activated ECs under inflammatory stimulation and typically recognizes glycoprotein ligands, among which the best-characterized ligand is P-selectin glycoprotein ligand-1 (PSGL-1) (24, 25). It plays a major role in the neutrophil-endothelial interactions, such as the initial capture and rolling of circulating neutrophils along the vascular wall, a process essential for leukocyte extravasation into inflamed tissues (26–28).

Zhang et al. found that the glycosylated cell surface RNA on neutrophils could specifically bind to endothelial P-selectin, and this interaction has significant immunological consequences (9). In this study, the interaction between neutrophil glycoRNA and ECs is verified to promote the physiological process of neutrophil recruitment and transendothelial migration, processes that are initiated and coordinated through selectin-mediated adhesive interactions under shear flow conditions (29). The importance of cell surface RNAs in neutrophil-endothelial interaction is examined through intravital imaging on mice pretreated with tumor necrosis factor alpha. The removal of neutrophil cell surface RNAs by extracellular RNaseA significantly impaired neutrophil capture and rolling on the endothelium in blood vessels, while stable adhesion remained unaffected. Subsequently, glycoRNA was shown to specifically bind to endothelial P-selectin. Importantly, blocking antibodies against P-selectin significantly reduced glycoRNA binding, whereas inhibition of E-selectin, another main lectin known to be expressed on ECs, did not affect this interaction. Genetic approaches further strengthened this conclusion: endothelial cells lacking P-selectin, generated through genetic knockout of the SELP gene, exhibited an obvious decrease in glycoRNA binding compared to wild-type controls. Together, these results support a model in which glycoRNAs function as previously unrecognized selectin ligands that cooperate with canonical glycoproteins to regulate leukocyte-endothelial interactions.

As one of the first discovered biological functions of glycoRNAs, the identification of P-selectin as a receptor for glycoRNAs highlights an unexpected mechanism through which cell surface RNAs can participate in immune regulation. By acting as adhesion ligands on the cell surface, glycoRNAs expand the molecular repertoire of selectin-binding molecules and introduce RNA-based structures into the framework of immune cell recognition. It also should be noted that, definitive biophysical or structural evidence directly characterizing glycoRNA-P-selectin interaction at the molecular level remains to be established.

### 3.2 Siglec and immune tolerance modulation

As another prominent member of the lectin family, Siglecs (sialic acid-binding immunoglobulin-type lectins) represent a collective group of homologous receptors on the cell surface that specifically bind to sialic acid-containing ligands (30, 31). To date, 14 Siglec members have been identified in humans, most of which are expressed on immune cells (32). They play a critical role in distinguishing self from non-self and regulate diverse immune cell functions in the innate and adaptive immune system by binding to their respective glycan ligands (33, 34). Recently, emerging evidence indicated that, in addition to their canonical ligands such as glycoproteins and glycolipids, certain Siglecs may also recognize glycoRNAs. Among them, Siglec-5, Siglec-11, and Siglec-14 have been implicated in glycoRNA binding (1, 10, 19).

The first example is that Flynn et al. reported the discovery of cell surface glycoRNA and proved that Siglec receptors can bind cell surface glycoRNA (1). Flow cytometry analysis using 12 different Siglec-Fc probes revealed that Siglec-11 and Siglec-14 exhibited RNase-sensitive binding to the cell surface, supporting glycoRNAs as potential ligands for these receptors. Building on this foundation, Chai et al. more explicitly demonstrated that Siglec-11 binds cell surface glycoRNAs in an RNase-sensitive manner (19). Through co-localization analysis using an anti-double-stranded RNA antibody, the authors showed a strong spatial correlation between Siglec-11 and glycoRNA on the cell surface, in contrast to other Siglecs such as Siglec-7 and Siglec-9. Next, biochemical assays were conducted, in which immunoprecipitation of Siglec-11, followed by glycoRNA labeling, demonstrated that Siglec-11 can directly enrich sialylated glycoRNAs. In addition, Li et al. identified Siglec-5 as a receptor for immune cell surface glycoRNAs. The authors discovered that the glycoRNA-Siglec-5 interaction mediates monocyte-endothelial adhesion, as disruption of cell surface RNA or blockade of Siglec-5 significantly reduced monocyte adhesion (10). To investigate receptor recognition, binding assays with Siglec-Fc fusion proteins were performed, demonstrating that Siglec-5 interacts with cell surface glycoRNAs. Importantly, this interaction was markedly reduced following RNase treatment, indicating that Siglec-5 binding is dependent on RNA integrity. Further biochemical analyses, including immunoprecipitation, supported a direct association between Siglec-5 and glycoRNAs. These comprehensive findings suggest that glycoRNAs act as previously unrecognized adhesion ligands and implicate the glycoRNA-Siglec-5 axis in inflammatory cell recruitment.

Collectively, these studies establish glycoRNAs as a novel class of ligands for certain Siglec receptors and expand the current understanding of Siglec-mediated immune recognition beyond traditional pathways. While the initial discovery revealed the existence of glycoRNA-Siglec interactions, subsequent work has begun to uncover their functional relevance in immune regulation, including roles in cell adhesion and signaling modulation. Nevertheless, the molecular mechanisms underlying these interactions, their broader immunological implications, and the extent to which other Siglec family members can recognize glycoRNAs remain to be fully elucidated.

### 3.3 Toll-like receptors and nucleic-acid sensing

Toll-like receptors (TLRs) are essential pattern recognition receptors of the innate immune system, detecting pathogen-associated molecular signatures to initiate inflammatory and antiviral responses. Among the TLR family, the endosomal nucleic acid-sensing subgroup (e.g., TLR3, TLR7, and TLR8) plays a particularly vital role in discriminating foreign from self-RNA. TLR3 recognizes double-stranded RNA through TRIF-dependent interferon regulatory factor 3 (IRF3) activation (35–37), while TLR7 and TLR8 detect single-stranded RNA catabolites generated by the lysosomal endoribonuclease RNase T2, with free uridine serving as an obligate co-ligand (38, 39). Since endogenous RNA is abundantly uridine-rich, active mechanisms must prevent self-sensing. Dysregulation of nucleic acid-sensing TLR pathways is clinically consequential, as dramatically illustrated by gain-of-function TLR7 variants sufficient to drive human systemic lupus erythematosus (40, 41). Karikó and colleagues established that naturally incorporated nucleoside modifications (pseudouridine, m^6^A, and m^5^C) potently suppress TLR3, TLR7, and TLR8 activation, demonstrating that chemical decoration of self-RNA is central to immune tolerance (42, 43). Building directly on this discovery, Flynn and Rathinam lab demonstrated that N-glycan modifications on glycoRNAs serve a protective function by shielding the immunostimulatory nucleoside 3-(3-amino-3-carboxypropyl) uridine (acp^3^U) from endosomal TLR3 and TLR7 recognition (2). Removal of N-glycans from the surface of apoptotic cells by the PNGase F induces significant type I interferons β (IFNβ) production in these cells after phagocytosis by macrophages, ultimately triggering immunostimulation. Furthermore, the RNA of DTW Motif TRNA-Uridine Aminocarboxypropyltransferase 2 (DTWD2)-knocked-out cells loses its immunostimulatory activity even after deglycosylation, indicating that the acp^3^U base is required for TLR3/7 activation. Together, these findings broaden our understanding of how cells maintain immunological tolerance to self-RNA and suggest that disruption of RNA glycosylation homeostasis may represent a previously unappreciated pathway to endosomal TLR-driven autoinflammation.

### 3.4 Other cell surface glycorna-binding proteins and immune recognition

Cell surface proteins are essential mediators of immune surveillance and cellular communication (44, 45). The recent characterization of glycoRNA and cell surface RNA-binding proteins (csRBPs) has unveiled a novel dimension of cell-surface biology. Rather than being passive or transient anomalies, these macromolecules organize into distinct nanoclustered domains that actively regulate intercellular communication, signal transduction, and molecular entry (15, 19, 46).

The presentation of csRBPs represents a critical and multi-layered platform for immune system modulation. Recent breakthroughs have revealed that these csRBPs co-assemble with glycoRNA to form specific nanoscale cluster domains, serving as a critical gateway for the entry of the prototypical cell-penetrating peptide (CPP) trans-activator of transcription (TAT) into cells (15). This RNA-dependent import mechanism implies that csRBPs-glycoRNA clusters function as a regulated gateway at the plasma membrane.

In addition, the therapeutic potential of aberrant csRBPs-glycoRNA presentation is powerfully illustrated in acute myeloid leukemia (AML). Another study identified specific csRBPs as AML-selective surface antigens absent from normal hematopoietic stem and progenitor cells. George et al. characterized nucleophosmin (NPM1) as an abundant csRBP uniquely displayed on AML blasts and leukemic stem cells within glycoRNA-csRBP nanodomains but absent from normal hematopoietic stem cells. Targeting these tumor-specific csRBPs-glycoRNA clusters with monoclonal antibodies has demonstrated profound preclinical success, effectively eliminating leukemic stem cells without harming normal hematopoietic tissues (47). While the clinical efficacy of cancer immunotherapies often sacrifice from a lack of target specificity, the discovery of precisely partitioned RBP-glycoRNA complexes provides a novel class of tumor-specific surface markers for AML treatment.

The therapeutic promise of glycoRNA-csRBP clusters further motivates a deeper inquiry into how these extracellular RNA-protein complexes interface with the broader cell-surface environment to influence immune recognition. Li et al. demonstrated that csRNAs form a stable ternary complex with heparan sulfate (HS) proteoglycans and csRBPs, wherein csRBPs serve as essential adapter molecules that bridge the two otherwise electrostatically incompatible, negatively charged biopolymers (20). Researchers overcame the limitations of traditional live-cell RNA labeling by repurposing the immune system’s natural single-stranded RNA sensor, TLR7, into an innovative probe capable of specifically recognizing RNA on the surface of living cells. Relying on this probe, the team conducted a genome-wide CRISPR-Cas9 knockout screen coupled with flow cytometry, discovering that key enzymes in the HS biosynthesis pathway—including Exostosin Glycosyltransferase 2 (EXT2) and Heparan Sulfate 6-O-Sulfotransferase 1 (HS6ST1)—are essential for maintaining the stable presentation of csRNAs. Furthermore, based on Spatially Selective Crosslinking and Orthogonal Organic Phase Separation (SSCOOPS), this study utilizes the covalent crosslinking of RNA with adjacent binding proteins, followed by organic phase separation, to specifically extract RNA-protein complexes localized to the outer leaflet of the cell membrane. In the context, HS-associated csRNAs (hepRNAs) recruit the killer cell immunoglobulin-like receptor 2DL5 (KIR2DL5) to the tumor cell surface by functioning as a co-receptor that concentrates KIR2DL5 locally and compensates for the inherently weak direct affinity between KIR2DL5 and its protein ligand Poliovirus Receptor. This establishes a mechanism by which surface RNA abundance can directly titrate inhibitory checkpoint engagement.

Building on the established role of heparan sulfate in stabilizing csRNAs-csRBP interactions, researchers have extended this framework beyond immune checkpoint regulation to encompass broader extracellular signaling contexts. Recently, Chai et al. elucidated the structural basis for this regulation through genome-wide CRISPR screening, demonstrating that HS proteoglycans act as fundamental organizers of cell surface glycoRNA-csRBP clusters (19). Specifically, the 6-O-sulfation of HS chains is the critical determinant for assembling these clusters, providing a direct mechanistic link between the HS and the spatial organization of the RNA-protein surfaceome. Once assembled, these glycoRNA-heparan structures serve as direct regulators of angiogenesis by binding to the heparan sulfate-binding domain of vascular endothelial growth factor A (VEGF-A). Surprisingly, these surface RNA clusters antagonize heparan sulfate-mediated activation of downstream extracellular regulated protein kinases (ERK) signaling. Mechanistically, abundantly enriched glycoRNA imposes precise negative constraints on the signaling cascade by competitively occupying the canonical HS-binding domain of VEGF-A_165_, which concurrently possesses direct RNA-binding capabilities. Consequently, disrupting the interaction between surface RNA and VEGF-A enhances intracellular ERK activation and impairs vascular development across both *in vitro* cell lines and *in vivo* zebrafish models. The above studies demonstrate that the glycoRNA-HS axis participates in the regulation of growth factor signaling, suggesting that cell-surface RNA complexes function as generalizable rheostats across diverse extracellular signaling contexts.

## 4 Conclusions and future perspectives

The discovery of cell surface glycoRNAs has expanded the conceptual framework of immune recognition by revealing RNA-containing glycoconjugates as previously unrecognized ligands at the cell surface. GlycoRNAs can participate in immune interactions through multiple molecular interfaces. On one hand, their sialylated glycan structures enable recognition by classical glycan-binding immune receptors such as selectins and Siglecs. On the other hand, their RNA backbones, together with associated RBPs and HS-containing proteoglycans, create composite molecular assemblies that may engage additional recognition pathways, including nucleic acid-sensing receptors. Through these diverse interfaces, glycoRNAs may contribute to key immunological processes such as leukocyte recruitment, immune tolerance, inflammatory signaling, and intercellular communication.

Despite these emerging insights, many questions remain regarding how glycoRNAs shape immune responses. A central challenge will be to determine the relative contributions of glycan-mediated recognition and RNA-dependent signaling in glycoRNA-immune receptor interactions. In addition, the mechanisms by which glycoRNA-RBP-HS assemblies regulate receptor engagement, signaling specificity, and immune cell activation remain largely unexplored. Another major challenge is that, although multiple complementary approaches have been developed to profile glycoRNA-protein interactions, direct structural and biophysical evidence demonstrating glycoRNA binding to its cognate ligands remains largely lacking. This limitation is partly attributable to the inherent technical challenges of applying cryo-electron microscopy to glycoRNA complexes, whose structural heterogeneity and conformational flexibility complicate high-resolution structural determination. In addition, detailed characterization of receptor-ligand binding is recommended, with experiments such as bio-layer interferometry (BLI), surface plasmon resonance (SPR). Nevertheless, the emerging evidence for glycoRNA interactions with immune receptors summarized in this Review represents a substantial advance in our understanding of glycoRNA-mediated immune recognition and provides an important framework for future mechanistic investigations.

Future studies integrating chemical biology, immunology, and glycobiology will be critical for defining the immunological roles of glycoRNAs in physiological and disease contexts. A deeper understanding of glycoRNA-mediated immune recognition may reveal new principles of immune regulation and provide opportunities for developing glycoRNA-based biomarkers or therapeutic strategies targeting immune signaling pathways.
